# A Sweet Spot for Molecular Diagnostics: Coupling Isothermal Amplification and Strand Exchange Circuits to Glucometers

**DOI:** 10.1038/srep11039

**Published:** 2015-06-08

**Authors:** Yan Du, Randall A. Hughes, Sanchita Bhadra, Yu Sherry Jiang, Andrew D. Ellington, Bingling Li

**Affiliations:** 1 Center for Systems and Synthetic Biology, The University of Texas at Austin, Austin, TX 78712, USA; 2 Applied Research Laboratories, The University of Texas at Austin, Austin, TX 78758, USA; 3 Department of Chemsitry, The University of Texas at Austin, Austin, TX 78712, USA; 4 Present address: State Key Laboratory of Electroanalytical Chemistry, Changchun Institute of Applied Chemistry, Chinese Academy of Sciences, Changchun, Jilin, 130022, P.R. China

## Abstract

Strand exchange nucleic acid circuitry can be used to transduce isothermal nucleic acid amplification products into signals that can be readable on an off-the-shelf glucometer. Loop-mediated isothermal amplification (LAMP) is limited by the accumulation of non-specific products, but nucleic acid circuitry can be used to probe and distinguish specific amplicons. By combining this high temperature isothermal amplification method with a thermostable invertase, we can directly transduce Middle-East respiratory syndrome coronavirus and Zaire Ebolavirus templates into glucose signals, with a sensitivity as low as 20–100 copies/μl, equating to atto-molar (or low zepto-mole). Virus from cell lysates and synthetic templates could be readily amplified and detected even in sputum or saliva. An OR gate that coordinately triggered on viral amplicons further guaranteed fail-safe virus detection. The method describes has potential for accelerating point-of-care applications, in that biological samples could be applied to a transducer that would then directly interface with an off-the-shelf, approved medical device.

The Middle-East respiratory syndrome coronavirus (MERS-CoV) belongs to the human betacoronavirus family of coronaviruses, which are large positive-stranded RNA viruses whose genomes typically range between ~27 to ~31 kb in size. The first case of MERS-CoV infection was identified in 2012 in a Saudi Arabian patient who died from a severe respiratory illness^1^,^2^,^3^. Since then, the infection has spread globally and through the middle of 2014 had caused 614 laboratory-confirmed cases and 181 deaths^2^,^4^,^5^. MERS-CoV is thus one of the most serious emergent viral pathogens since its relative, SARS-CoV^6^.

In order to control spreading infections and receive timely treatment, sensitive and specific detection of MERS-CoV at point-of-need or point-of-care is extremely important. While methods such as immunofluorescence assays (targeting MERS-CoV N and S proteins)^7^ and real-time reverse transcription polymerase chain reaction (RT-PCR, targeting viral genes in or around ORF1A, ORF1B and upE) have been successfully used for clinical diagnosis^8^,^9^,^10^, these methods are not readily adapted to point-of-care applications. A simple molecular assay for MERS-CoV can potentially be developed by relying on the exquisite sensitivity and extreme simplicity of isothermal amplification reactions^11^,^12^,^13^,^14^,^15^,^16^, such as loop-mediated isothermal amplification (LAMP), which yields up to 10^10^-fold amplification within 10 min to two hours^17^,^18^. Unfortunately, LAMP also creates many non-specific amplicons that often yield false positive results and because of this its potential for point-of-care assays has not been realized.

We recently found that sequence-specific, nucleic acid strand exchange reactions can be used to efficiently distinguish between specific and non-specific LAMP amplicons^19^,^20^,^21^. We now show that LAMP reactions can be made user-friendly and field-based by integrating LAMP, strand exchange signal transduction, and a commercial-off-the-shelf device, a glucometer, for pathogen detection. The use of glucometers to monitor non-glucose targets was pioneered by Lu’s group, who adapted this device for aptamer- or DNAzyme-based detection of cocaine, ATP and metal ions^22^,^23^,^24^,^25^. These and other efforts^26^,^27^,^28^,^29^,^30^ inspired our more generalizable approach to molecular diagnostics. The addition of a thermostable invertase allows all reactions to occur in the same tube at a wide temperature range, and the simplicity of the format ultimately yields atto-molar (or low zepto-mole) detection of pathogens such as MERS-CoV or Zaire Ebolavirus (ZEBOV or Zaire Ebola), even in sputum or saliva samples. The strand exchange reactions ensure low false positives, while the use of an OR gate guards against false negative detection. Moreover, the reaction components are modular and can be readily adapted to different protocols covering different reaction temperatures, times, volumes, and vagaries of reagent storage.

## Results

### Adapting isothermal amplification to a glucometer

**Figure 1:**
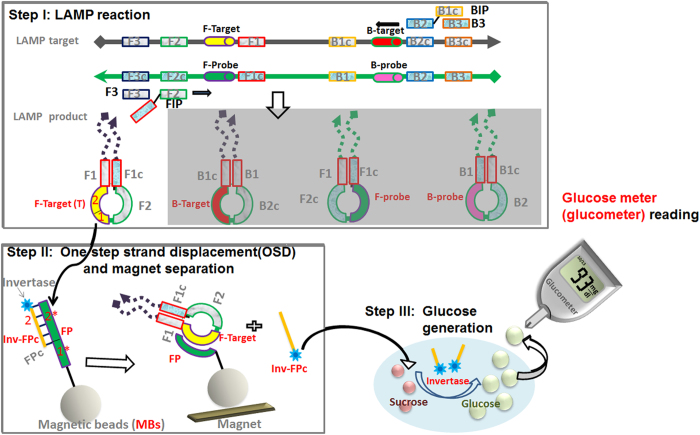
Scheme for adapting isothermal amplification to a glucometer. The detection scheme includes three steps: **i**) isothermal LAMP amplification of the target viral RNA/DNA produces flower-like amplicons containing four kinds of single-stranded loops, positioned between the target loci F2-F1 (F-target), F2c-F1c (F-probe), B2-B1 (B-probe), and B2c-B1c (B-target), respectively; **ii**) one of the LAMP loops (e.g., F-target) initiates a one-step strand displacement (OSD) reaction to release the Inv-FPc reporter sequence from FP conjugated to magnetic beads; **iii**) after magnetic separation, the released invertase on Inv-FPc reporter produces glucose and is read via a glucometer. F3: forward outer primer, B3: reverse outer primer, FIP: forward inner primer, BIP: reverse inner primer.

Our process of adapting isothermal amplification to the glucometer can be divided into three steps (Fig. 1): i) isothermal amplification; ii) strand displacement to release invertase and separation of the released invertase; iii) production of glucose by the released invertase and readout via a glucometer.

A synthetic segment of MERS-CoV genome (corresponding to sORF1A^10^, was initially used as a model analyte. A standard LAMP reaction was carried out to produce flower-like amplicons that had four kinds of single-stranded loops, positioned between F2-F1 (F-target), F2c-F1c (F-probe), B2-B1 (B-probe), and B2c-B1c (B-target), respectively (Fig. 1, Step I). In the second step (Fig. 1, Step II), LAMP amplicons were mixed with magnetic beads containing a hemiduplex oligonucleotide strand displacement probe (OSD) that was constructed by hybridizing an oligonucleotide complementary to the F-target (FP) with an antisense strand conjugated to invertase (Inv-FPc). The F-target itself (domain 2-1 in Fig. 1) is localized within an amplicon loop. It can initiate strand displacement of the OSD by binding to toehold domain 1* on FP, ultimately leading to the displacement of Inv-FPc (domain 2). After magnetic separation of the beads, the displaced Inv-FPc hydrolyzes sucrose into the monosaccharides glucose and fructose (Fig. 1, Step III) and the resulting glucose is then detectable by using any commercial blood glucose meter (Fig. 1, Step III).

The steps were initially validated separately. A 5-primer LAMP reaction for sORF1A had previously been developed in our laboratory^20^ and was found to detect as few as 5 to 50 PFU/ml of MERS-CoV in infected cell culture supernatants (Step I). Yeast invertase was conjugated to FPc^22^ and could still successfully hydrolyze sucrose at its optimum temperature, 55 °C (Figure S1A). While we directly hybridized the invertase conjugates to beads, initial purification of the DNA-invertase conjugates prior to hybridization may further improve signal generation^25^. The efficiency of strand displacement (Step II) was assessed using DNA mimic of the LAMP loop (1A-T). Strand displacement of an invertase conjugate was measured as a function of 1A-T concentration, and as little as 1 nM of oligonucleotide could be detected by a glucometer after 40 min (Step III, Figure S2). We anticipated that LAMP should produce at least 5 nM each amplicon loops to initiate strand displacement.

### Demonstrating LAMP-to-glucose transduction

Following the demonstration of individual components, the three steps were integrated for MERS-CoV detection. Initial integration was via sequential transfer between different reservoirs. This was in part because the temperatures required for each step were different: both LAMP and glucose production worked at 55 °C, while OSD and magnetic separation were at room temperature. The LAMP amplicons (10 μL) were mixed with Inv-FPc/FP modified magnetic beads (18 μg) and incubated for 1 hour prior to magnetic separation and transfer of the supernatant into a 500 mM sucrose solution. After 40 minutes, glucose produced by the action of the released invertase was read using the glucometer.

**Figure 2:**
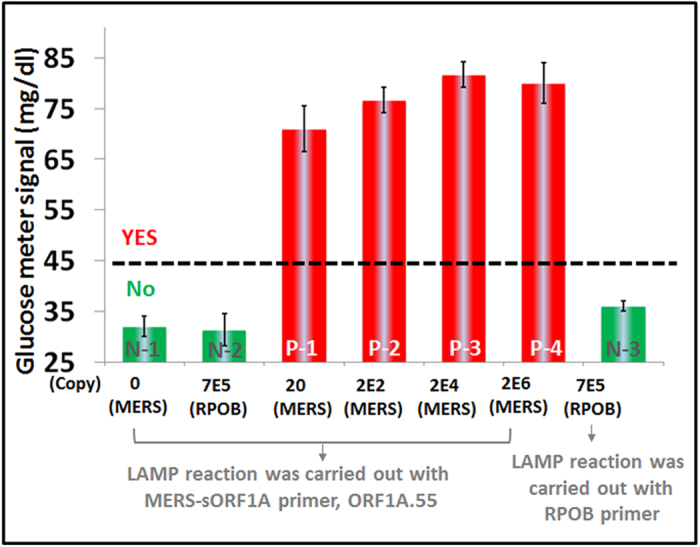
Sensitive and specific detection of synthetic MERS-CoV DNA (sORF1A) using LAMP-to-glucose transduction. The bar-graph presents glucometer responses to both non-target (RPOB) and different amounts of target (MERS-CoV sORF1A = MERS) templates. N-1, N-2, P-1 to P-4 were amplification reactions with the ORF1A.55 MERS-CoV primer set. Cognate targets (P) respond while non-cognate targets or negative controls (N) do not. Assays included a 1.5 hour 55 ^o^C LAMP reaction, 1 hour 25 ^o^C OSD, and 40 min 55 ^o^C glucose generation using a commercial yeast invertase (yeast INV). The error bars represent standard deviations calculated from three parallel assays.

This sequential processing led to the definitive detection of a synthetic template representing sORF1A (at least 35 mg/dl or higher glucometer signals) relative to negative controls (isothermal amplification buffer with no sORF1A template or non-cognate templates, such as *M. tuberculosis rpoB* genes, RPOB) (Fig.2 and Figure S3). While as few as 20 copies of the template could be detected, there was no dose-discrimination, as might have been expected for a robust amplification reaction such as LAMP. In given reaction time, primers in all reactions have been consumed.

### Adapting LAMP-to-glucose transduction to RNA detection with thermostable invertase

**Figure 3:**
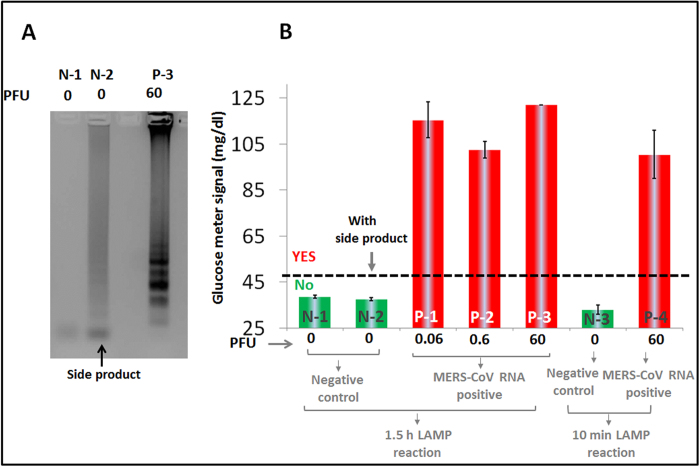
Detection of MERS-CoV RNA from tissue culture-derived virions through reverse transcription and LAMP amplification (RT-LAMP). (**A**) Agarose gel electrophoretic characterization of RT-LAMP amplicons from samples containing RNA extracted from 60 PFU of MERS-CoV virons (or 2 μL 3E4 PFU/mL) (P-3) or samples lacking MERS-CoV RNA (N-1 and N-2). For each sample, the LAMP reaction was carried out at 55 ^o^C for 1.5 hours and developed on a 1% agarose gel stained with ethidium bromide. (**B**) Glucometer responses for amplification reactions with RNAs extracted from 0.06 (P-1), 0.6 (P-2), 60 (P-3 and P-4) PFU MERS-CoV virions (2 μL) and with RNA-negative samples (N-1, N-2, and N-3). Samples underwent 1.5 hour or 10 min 55 ^o^C RT-LAMP reactions, 1 hour 25 ^o^C OSD, and 23 min 55 ^o^C glucose generation with thermostable TmINV. The signals shown in N-1, N-2 and P-3 in Fig 3B were obtained from the same RT-LAMP reactions as the N-1, N-2, and P-3 samples shown in Fig 3A. It should be noted that the LAMP reaction for sample N-2 produces non-specific amplicons but these do not produce a false positive glucometer signal. The error bars represent standard deviations calculated from three parallel assays.

In order to detect MERS-CoV itself, rather than a synthetic template, the basic integrated protocol had to be modified by introducing a reverse transcription step as a precursor to the LAMP reaction. The optimized RT-LAMP allowed the direct amplification of RNA isolated from Trizol-inactivated MERS-CoV (Jordan n3/2012 strain)-infected Vero cell culture supernatants (Fig. 3). These nucleic acid samples contained both MERS-CoV virions along with defective interfering (DI) particles (MERS-CoV RNA). Nonetheless, MERS-CoV RNA was detected specifically relative to negative controls, and any parasitic amplicons that were randomly generated during RT-LAMP did not yield false positive responses (Fig. 3A, N-2 and Fig 3B, N-2). Surprisingly, the signal intensity increased to ~60 mg/dl (compare with Fig. 2A), even though less time was allowed for glucose generation (23 min vs 40 min, Step III).

**Figure 4:**
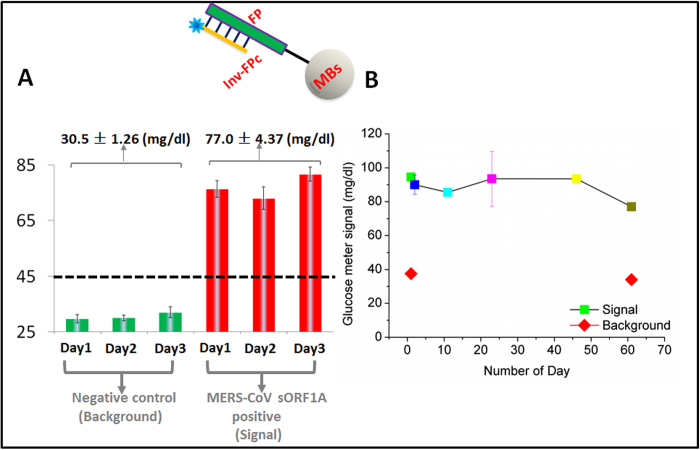
Stability and reproducibility of modified magnetic beads. (**A**) LAMP-to-glucose assays with 2E4 copies of MERS-CoV sORF1A target DNA (Day 1, Day 2, and Day 3, red) and buffer controls (Day 1, Day 2, and Day 3, green), carried out with conjugated magnetic beads (Inv-FPc/FP/MBs) that were prepared on three different days. On each day, three parallel assays were carried out with both the target MERS-CoV sORF1A DNA and buffer controls. A total of nine assays were performed for both the sORF1A target and buffer controls. Assay conditions were otherwise the same as in Fig 3. (**B**) Time dependence of glucometer responses to LAMP amplicons generated from 2E4 copies of sORF1A using a single preparation of Inv-FPc/FP/MBs. The error bars represent standard deviations calculated from three parallel assays. The commercial yeast INV was used in these experiments.

The improved performance could be explained in part by the fact that we switched from a mesophilic yeast invertase to a higher performance thermostable invertase derived from the hyperthermophilic bacteria, *Thermotoga maritime* (TmINV). By using TmINV we were able to detect as few as 0.06 plaque forming units (PFU) or 2 μL 30 PFU/mL (in each 25 μL LAMP reaction) of MERS-CoV virions in infected cell culture supernatants with a 1.5 hour LAMP reaction. This was in turn estimated to be about 100 copies of RNA based on real-time reverse transcription PCR (Figure S4)^7^,^8^,^20^,^31^. Moreover, assays performed with a much shorter LAMP reaction (10 rather than 90 min) suggested that the signal-to-noise was more than adequate to detect the virus (Fig. 3B, N-3 and P-4).

Moreover, in order to ensure that the programmed molecular interactions would potentially function in an off-the-shelf device, we determined the reproducibility of assays following preparation and storage of the reagents. Initial reproducibility assays were carried out by comparing assays in triplicate using Inv-FPc/FP/MBs that were prepared on three consecutive days (Fig. 4A). As expected, very small deviations were seen between all nine sets of measurements (1.26 mg/dl and 4.37 mg/dl for negative and positive responses, respectively, both of which were below 6% of their average signals). We further tested the stability of Inv-FPc/FP/MBs by measuring responsivity to 2E4 copies of sORF1A every 2 to 20 days (Fig. 4B). Reproducible performance could be observed over at least 60 days when the reagents were stored at 4 °C.

### Integrating LAMP-to-glucose detection in one tube with thermostable invertase.

To begin to integrate amplification with detection in one reaction and on one platform, we developed two additional methods (Protocol 2 and Protocol 3) in which OSD was carried out at 55 °C rather than at room temperature (25 °C). The toehold in the FP strand was designed with 10 nucleotides in order to ensure robust interactions with the F-target in the LAMP loops, even at higher temperatures. Initially, we just repeated the method described above (Protocol 1) in which amplification, separation, and glucose generation steps were carried out separately and aliquots were transferred, except that all steps were now at 55 °C (Protocol 2, using thermostable invertase). Glucose was again read as an end-point of the assay, and a positive signal (with similar signal amplitude over negative control as Protocol 1) could be read after only 11 minutes, with MERS-CoV RNA extracted from 60 PFU MERS-CoV virus. There was slightly higher background at the higher temperature, and the overall signal:noise ratio decreased by a factor of two (Figure S5).

These results encouraged the development of an even simpler method in which the amplification and strand displacement steps occurred in the same tube (Protocol 3, using thermostable invertase). Following the transfer of released thermostable invertase from magnetic beads (and still-bound invertase), the addition of sucrose can again lead to glucose development. The signal, background, and signal-to-noise ratio for this method were similar to those seen for Protocol 2 (Figure S5). Even though Protocol 3 is a two-stage protocol and not a real-time signaling method such as real-time PCR, it shortens detection relative to the three-stage Protocol 1 and Protocol 2.

The use of thermostable invertase was critical for the development of the one tube assay. Although the mesophilic yeast invertase is more commonly used^22^,^23^,^24^,^26^, it is only stable in the presence of its substrate, sucrose, and loses activity after only 3 min of incubation alone at 55 ^o^C (Figures S1B and S1C). While sucrose is commonly used for enzyme stabilization^32^,^33^,^34^, its inclusion would obviously be problematic for the transduction mechanism we have proposed.

### LAMP-to-glucose transduction can be readily adapted to different molecular targets.

**Figure 5:**
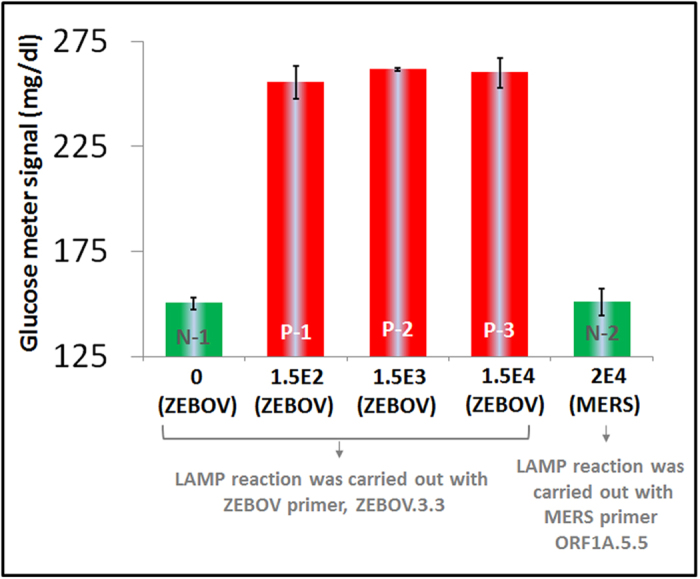
Detection of synthetic ZEBOV DNA (ZEBOV-VP30) using LAMP-to-glucose transduction. Glucometer responses to 1.5E2 copies (P-1), 1.5E3 copies (P-2) and 1.5E4 copies (P-3) of synthetic ZEBOV VP30 (= ZEBOV) or to a DNA buffer control (0 copies, N-1). N-2 is the negative control for OSD signal transduction reaction and shows no response even in the presence of an amplification reaction seeded with 2E4 copies of the non-cognate MERS-CoV sORF1A (= MERS) template and appropriate MERS-specific primers. Reaction conditions were as in Fig 3. The error bars represent standard deviations calculated from two parallel assays. The thermostable TmINV was used in these experiments.

Using the same methods that were worked out for MERS-CoV, it proved possible to immediately develop amplification reagents and probe sets for the Zaire Ebolavirus (ZEBOV or Zaire Ebola) virus. A synthetic segment of ZEBOV genome (corresponding to a portion of the VP30 gene, ZEBOV VP30) was chosen as an analysis template, and primers were designed that yielded specific and robust amplification. The FP/Inv-FP duplex (for MERS-CoV) on magnetic beads was swapped with a probe set for ZEBOV VP30 (ZEBOV-FP/Inv-ZEBOV-FPc) that released Inv-ZEBOV-FPc only in response to a F-target loop in an ZEBOV VP30 amplicon. As shown in Fig 5, as few as 150 copies of this template displayed a glucometer signal that was at least 90 mg/dl than the negative buffer control (N-1) or non-specific templates (MERS-CoV sORF1A, N-2). As with MERS-CoV, a molecular amplifier and transducer that could potentially be used as a field test for the ZEBOV virus can be readily envisioned based on these results.

### Molecular computation improves the robustness of sensing.

One of the key problems with any molecular assay is false signals, either false negatives or false positives. This is especially true for isothermal amplification reactions, in part because of non-specific amplification background that can yield false positive signals, and in part because complex amplification reactions can fail and thereby yield false negative signals. Strand exchange DNA circuitry and nano-technology has been proven very helpful in realizing more robust and intelligent reaction and signaling^35^,^36^,^37^. We have demonstrated that OSD transduction and a previously developed AND gate can be used to guard against false positives^19^. Now we show how an OR gate can guard against false negatives induced by inadvertent failure of a LAMP reaction.

**Figure 6:**
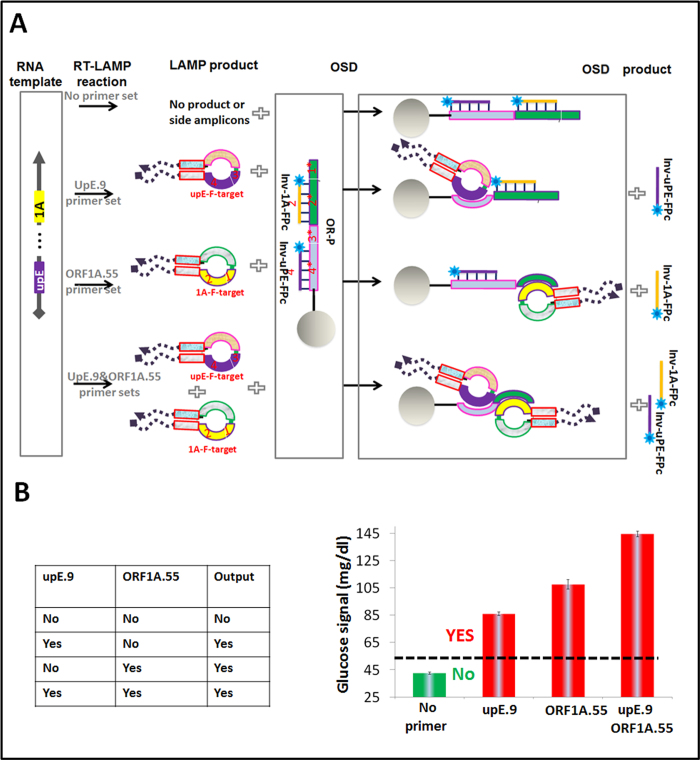
Using an OR gate to improve the robustness of sensing. (**A**) OR gate design. Either the ORF1A region or the upE region of amplicons produced from a MERS-CoV template could initiate OSD. The respective amplicons will displace either an Inv-ORF1A-Fc or an Inv-upE-FPc specific reporter strand bound to the complementary OR-P capture strand attached to magnetic beads. Either or both released invertases will lead to a glucometer response. (**B**) Performance of the OR gate with RNA extracted from 60 PFU MERS-CoV virons amplified with either no primers, with the upE.9 and ORF1A.55 individually, or with a combination of the upE.9 and ORF1A.55 primers. Reaction conditions were as in Fig 3. The error bars represent standard deviations calculated from two parallel assays. The thermostable TmINV was used in these experiments.

A strand exchange OR gate was developed that responds to either one of two LAMP products, one amplified from the ORF1A region or the other from the upE region on MERS-CoV RNA. Briefly, F-target loops amplified from either the ORF1A region (1A-F-target) or the upE region (upE-F-target) were designed to bind to domains 1* or 3* on OR-P, respectively, and thereby trigger one of two different toehold-mediated strand displacement reactions (displacing, respectively, Inv-1A-FPc or Inv-upE-FPc away from OR-P) (Fig. 6A).

This molecular scheme was first implemented with upE-T and 1A-T, two linear oligonucleotides that mimicked the MERS target. Both oligonucleotides yielded a positive response individually, and together they showed almost double the positive signal (Figure S6). When MERS-CoV RNA was used as input either or both inputs again yielded a positive signal (Fig. 6B).

## Discussion

One of the key issues with the development of point-of-care or point-of-need diagnostics, especially in resource-poor settings, is the lack of sophisticated equipment to carry out measurements and the lack of sophisticated users to interpret those measurements. We therefore decided to adapt molecular amplification assays to glucometers, a device that is almost ubiquitous, and whose interpretation has become standardized across many users. We show how an isothermal amplification reaction can lead to the production of amplicons that specifically strand exchange invertase, which in turn leads to the production of an easily read glucose signal from otherwise inert sucrose. The sensitivity of the assays was mainly provided by the powerful signal amplification properties of LAMP, while selectivity was provided by both the hybridization specificities of the LAMP primers and of the interaction of LAMP product loops with strand displacement reactions.

These technologies were applied to an important public health problem, the detection of viruses that have the capability to cause epidemics. We have successfully shown the assay could provide trustworthy “YES-or-NO” answers for the presence of even small amounts of MERS-CoV in a complex mixture. The sensitivities obtained were on the order of atto-molar (or low zepto-mole) ultra-sensitivity with either DNA or RNA templates. We then demonstrated that by simply changing primer and probe sequences the same assay could be easily reconfigured for the Zaire variant of the Ebola virus, with similar sensitivities.

While qPCR is a gold standard for molecular diagnostics, it remains difficult to implement in resource-poor settings and at point-of-need or point-of-care, and can still yield false-positive results (Figure S4). The isothermal amplification reactions and transducers we have developed are similarly sensitive, but also extremely robust to field conditions, including variations in temperature, time of incubation, volume, and reagent storage. We have investigated several different configurations of the assay, all of which have proven successful. We have investigated several different configurations of the assay, all of which have proven successful. The LAMP reaction can be carried out at 55 ^o^C ± 10 ^o^C. The OSD transducer and glucose generation can be carried out at any temperature between 25 ^o^C–65 ^o^C. Even though we 1.5 hour LAMP and 1 hour OSD reactions were used throughout we estimate that the integrated the detection of viral pathogens might be completed within as short as 25 min (10 min LAMP (Fig. 3B), 10 min OSD^21^, and 5 min glucose production). The total reaction volume may be compressed, although likely not to less than 20 μL. The most important reaction component, the invertase-DNA-magnetic bead assembly, can be stored at 4 ^o^C for at least 60 days without losing significant activity.

One issue with the transduction to glucose and readout by a glucometer is the potential presence of different levels of background glucose in biological samples. For more complex samples where endogenous glucose may affect virus detection, it may be possible to first remove background glucose or to do background subtraction by recording the ambient glucose level in the sample.^22^ But the effect of existed glucose will be of less import with the detection of MERS-CoV or ZEBOV in oral or other non-blood or non-urine samples. For example, viral loads in a MERS-CoV patient are as high as 1–2 × 10^6^ copies per mL in the lower respiratory tract, 1 × 10^3^ copies per mL in stool, and 5–6 × 10^3^ copies per mL in an oronasal swab^38^. It was reported that there should be even more detectable MERS-CoV virus in sputum than in oronasal swabs.^39^ Given the excellent limit of detection (0.06PFU or 100 copies) we have observed, these clinically relevant limits should be observed against a moderate glucose background. For example, the average level of glucose in fasting saliva in non-diabetics is around 6 mg/dl, which would show a “LO” symbol in the glucometer and is small relative to our ~60 mg/ml signal amplitude.^40^ Similarly, RT-PCR results^41^,^42^ suggest that ZEBOV can also be detected in low glucose samples such as saliva, stool, and tears during the acute period of illness. Thus, while glucose interference should be taken into account during assay development, it should not obscure reliable, robust detection of viral pathogens via our transduction method. We have proven the ZEBOV VP30 template can be detected when it is spiked into synthetic sputum (Figure S7A) and human saliva samples (Figure S7B). The synthetic sputum contained human genomic DNA, salmon sperm DNA, mucin, and other components (provided by the Program for Appropriate Technology in Health, PATH, Seattle, WA).^21^ The mucin was liquefied with 2% freshly made N-acetyl-L-cysteine and 1% sodium hydroxide prior to assay because it inhibited the Bst 2.0 polymerase. Human saliva was directly used after 10 min pre-heating (at 95 ^o^C). The LAMP-OSD-glucometer method showed true positives in both 26% sputum and 20% saliva samples (Figure S7), without losing any sensitivity compared to pure buffer (Fig. 5). That said, signal amplitudes were decreased, especially for low copy samples.

## Conclusion

Herein, we show that isothermal amplification methods and nucleic acid strand exchange reactions can be used to transduce MERS-CoV virions into signals that can be easily read by a commercial glucometer. As few as 20 nucleic acid templates or 0.06 PFU of inactivated virus could be detected. ZEBOV virus could be detected by simply reconfiguring the primers and probes used for transduction. The nucleic acid strand exchange reactions were particularly amenable to further development for point-of-care applications, in that they were modular, programmable, and relatively insensitive to temperature and storage. These features were further highlighted by demonstrating that false negative reactions could be avoided by programming an OR gate into the transducer.

## Methods

### Chemicals and materials

The Bayer Contour Next Blood Glucose Test Strips and Bayer Contour Next Blood Glucose Monitoring System were bought from Amazon.com and used for the tests in this work. Streptavidin-coated magnetic beads (MB, 1.5 μm in average diameter) were purchased from Bangs Laboratories Inc. (Fishers, IN, USA) and the Amicon Ultra-2 mL 30 K was purchased from Millipore Inc. (Billerica, MA, USA). Illustra MicroSpin G-25 micro columns were purchased from GE Healthcare Bio-Sciences Corp. (Piscataway, NJ, USA). Sulfosuccinimidyl-4-(N-maleimidomethyl) cyclohexane-1-carboxylate (sulfo-SMCC) was bought from Pierce Biotechnology (Rockford, lL, USA). Grade VII invertase from baker’s yeast (*S. cerevisiae*), tris(2-carboxyethyl)phosphine hydrochloride (TCEP), and other chemicals and solvents were purchased from Sigma-Aldrich, Inc. (St. Louis, MO, USA). All enzymes including *Bst* 2.0 DNA polymerase and AMV reverse transcriptase were obtained from New England Biolabs (Ipswich, MA, USA). MERS-CoV sORF1A DNA, RPOB DNA, and ZEBOV DNA templates were home-made. MERS-CoV-containing Vero cell culture supernatants were a gift from Dr. Reed Johnson at the National Institutes of Health. Thermostable invertase derived from the hyperthermophilic bacteria, *Thermotoga maritima,* was home made. All the oligonucleotides were purchased from Integrated DNA Technologies, Inc. (Coralville, IA) and listed in Table 1. The concentrations of the DNA suspensions were measured by UV spectrophotometry using the NanoDrop 1000 spectrophotometer (Thermo Scientific, Wilmington, DE, USA).

### Synthesis, Expression, and Purification of *Thermotoga maritima* Invertase

The amino acid sequence of the *Thermotoga maritima* MSB8 β-fructosidase (Invertase) was obtained from the National Center for Biotechnology Information (NCBI) database (GenBank # AAD36485.1). This sequence was reverse translated by using GeneDesign^43^ and codons were optimized for expression in *E. coli*. Additional 30 bp flanks were added to the ends of the gene to facilitate cloning via Gibson assembly^44^ into the pET21a (Novagen) expression vector. The TmINV gene was assembled from synthetic oligonucleotides according to the Protein Fabrication Automation methodology^45^. The assembled gene was cloned via Gibson assembly into the pET21a vector backbone with a C-terminal HisTag to obtain the sequence verified clone pET21-TmINV. The pET21-TmINV vector was transformed into BL21-AI competent cells (Invitrogen) for overexpression. The expression of TmINV was induced by adding 0.2% L-arabinose in 250 mL mid-log phase cultures of transformed BL21-AI grown in Superior Broth (Athena). The induced cultures were allowed to grow overnight at 24 °C. Following overnight expression the cells were lysed with 1 mg/mL chicken egg white lysozyme and sonication. The His-tagged TmINV protein was purified via immobilized metal affinity chromatography. The protein was dialyzed into 50 mM Sodium Phosphate Buffer, pH 7.4 containing 175 mM NaCl. Sample purity (indicated by the presence of a single ~51 kDa band) was verified by SDS-PAGE to be >98%. Concentration of the purified TmINV protein was determined via measurement of Abs280 nm (TmINV molar absorptivity = 86,080).

### Cloning of MERS-CoV and Zaire Ebolavirus VP30 gBlocks and PCR amplification of transcription templates (Making MERS-CoV sORF1A and ZEBOV V30 DNA templates)

All DNA polymerase chain reaction (PCR) amplification reactions were performed using high-fidelity Phusion DNA polymerase (NEB), according to the manufacturer’s instructions. The gBlock double stranded DNA surrogates of MERS-CoV and ZEBOV genetic loci were designed to include a T7 RNA polymerase promoter at their 5’-ends to enable subsequent transcription. These gBlocks were cloned into the pCR2.1-TOPO vector (Life Technologies, Carlsbad, CA, USA) by Gibson assembly using the 2x mastermix (NEB) according to the manufacturer’s instructions. Cloned plasmids were selected and maintained in an *E. coli* Top10 strain. Plasmid minipreps were prepared from these strains using the Qiagen miniprep kit (Qiagen, Valencia, CA, USA). All gBlock inserts were verified by sequencing at the Institute of Cellular and Molecular Biology Core DNA Sequencing Facility.

For performing *in vitro* run-off transcription, transcription templates cloned in a pCR2.1-TOPO vector were amplified from sequenced plasmids by PCR using Phusion DNA polymerase. Final PCR products (MERS-CoV sORF1A and ZEBOV V30) were verified by agarose gel electrophoresis and then purified using the Wizard SV gel and PCR Clean-up system, according to the manufacturer’s instructions (Promega, Madison, WI, USA).

Synthetic *M. tuberculosis rpoB* gene (RPOB) segment was generated from commercial genomic DNA of the virulent strain H37Rv (ATCC, Manassas, VA, USA) in the same protocol described above.

### Nucleic acid extraction (Extracting MERS-CoV RNA from tissue culture derived virons)

MERS-CoV viral RNA (MERS-CoV RNA) was prepared from infected Vero cell culture supernatants inactivated with 3:1 ratio of Trizol using the DirectZol RNA miniprep kit (Zymo Research, Irvine, CA, USA) according to the manufacturer’s protocol. The resulting viral RNA was used immediately and excess was stored at −80 °C. RNA was also extracted from similarly treated culture supernatants from uninfected Vero cells to serve as negative controls for the amplification assays.

### Synthesis of Inv-FPc conjugate

The Inv-FPc conjugate was synthesized according to a previously published protocol with slight modifications^22^. Invertase was conjugated to the linker by dissolving 2.5 mg of yeast invertase (or TmINV) and 1 mg of the sulfo-SMCC linker in 1 mL PBS buffer (10 mM Sodium Phosphate, 137 mM NaCl, 2.7 mM KCl, pH 7.4). This solution was shaken at 750 rpm for 2.5 hour at room temperature (RT, 25 °C). Subsequently, unreacted reagents were removed by filtration through an Amicon-30 K filter and the invertase-SMCC conjugate was subjected to six washes with PBS prior to resuspension in 850 μL PBS. Meanwhile, 120 μl of the thiol-labeled oligonucleotide SH-FPc (resuspended at a concentration of 125 μM in water) was activated for 1 hour at RT by continuously mixing with 15 μL of 100 mM TCEP. Excess TCEP and salts were subsequently removed by filtration through Sephadex G-25. Activated SH-FPc and the invertase-SMCC conjugate were then mixed and stirred overnight at 30 °C. Unreacted SH-FPc was removed by filtration through an Amicon-30 K filter and the Inv-FPc conjugate was subjected to at least six washes. The final Inv-FPc conjugate was resuspended in PBS buffer at a concentration of 5 mg/mL (determined with a Nanodrop ND-1000 Spectrophotometer) and stored at 4 °C until further use.

### Preparation of the Inv-FPc/FP/MB conjugate

Some 200 μL of 1 mg/mL streptavidin coated MBs were transferred into a 1.5 mL centrifuge tube and washed with isothermal amplification buffer (20 mM Tris-HCl, 10 mM (NH_4_)_2_SO_4_, 50 mM KCl, 2 mM MgSO_4_, 0.1% Tween 20, pH 8.8). The MBs were isolated by using an external magnetic rack and resuspended in 100 μL isothermal amplification buffer. Six microliters of 75 μM biotinylated oligonucleotides (biotin-FP, partial complement of FPc) were incubated with the MBs on a vertical rotator for 25 min. The unbound biotin-FP was removed by washing the FP/MBs in isothermal amplification buffer at least 5 times. Finally the FP/MBs were suspended in 50 μL isothermal amplification buffer and incubated for 1.5 hour with 10 μL of the 5 mg/mL Inv-FPc conjugates on a vertical rotator at RT. After at least five washes with 100 μL isothermal amplification buffer to remove excess Inv-FPc, the final Inv-FPc/FP/MBs (about 2 mg/mL) were dispersed in 100 μL isothermal amplification buffer and stored at 4 °C until further use.

### Detection of mimetic target with OSD-to-glucose transduction

A series of tubes containing 9 μL aliquots of the 2 mg/mL Inv-FPc/FP/MBs were placed close to the magnetic rack for 1 min. The clear solution was discarded and replaced by 10 μL of the mimetic target (1A-T, in isothermal amplification buffer) at different concentrations. The OSD reactions were allowed to proceed for 1 hour on a vertical rotator at RT. The Inv-FPc/FP/MBs were then separated using a magnetic rack and aliquots of the supernatant containing released Inv-FPc were transferred into equal volumes of 500 mM sucrose. This mixture was incubated for 40 min at 55 °C to allow invertase-mediated catalytic conversion of sucrose to glucose. Subsequently, 1 μL of the reaction solution was transferred to a glucometer strip and the amount of glucose was measured by using a commercially available hand-held glucometer.

### LAMP primer design

Design process of MERS-CoV LAMP primers is being published elsewhere. All viral genomic sequences were obtained from NCBI GenBank. All available ZEBOV genomic sequences were aligned using MUSCLE and the most conserved regions in the VP30 gene were used for designing LAMP primers. *PrimerExplorer 4* (Eiken) was used to generate 3 sets of LAMP primers composed of the outer primers F3 and B3 and the inner primers FIP and BIP. Primer design was constrained to include at least a 30 bp gap between the F1 and F2 as well as between the B1 and B2 priming sites. These gaps should become part of the LAMP amplicon loop structures and were included to allow the subsequent recognition by the OSD probes. Primer specificity for all the sequenced ZEBOV isolates and a corresponding lack of significant cross-reactivity to other nucleic acids of human, related filoviral or pathogenic origin was further assessed using NCBI BLAST. All three primer sets were tested by amplification of plasmids bearing ebolavirus genomic fragments in standard LAMP reactions. Amplicon accumulation was analyzed at end-point by agarose gel electrophoresis and in real-time by measuring the incorporation of the intercalating fluorophore EvaGreen. Ultimately one of the three primer sets, ZEBOV.3.3 that demonstrated target-dependent amplification kinetics and generated minimal spurious amplicons was chosen for further assay development.

### Standard LAMP reaction

Mixtures containing different copies of template (sORF1A), 1 μM each B1c-B2 and F1c-F2, 0.25 μM each B3 and F3, 0.5 μM LP, 1 M betaine, 2 mM MgCl_2_, and 0.4 mM dNTPs in a total volume of 24 μL 1 × Isothermal Buffer (20 mM Tris-HCl, 10 mM (NH_4_)_2_SO_4_, 10 mM KCl, 2 mM MgSO_4_, 0.1% Triton X-100, pH 8.8) were heated to 95 °C for 5 to 10 min, followed by chilling on ice for 2 min (this pre-denaturing process is not absolutely necessary but results in improved sensitivity). Then, 1 μL (8 U) of *Bst* DNA polymerase 2.0 was added to initiate the LAMP reaction. The reactions (with a final volume of 25 μL) were incubated for 1.5 hour in a thermal cycler maintained at 55 ^o^C. A 5 μl aliquot of the reaction mixed with 3 μl of 6 × DNA loading dye was then analyzed by electrophoresis through a 1% agarose gel containing ethidium bromide. Gel analysis of LAMP products was performed in a room completely separate from the normal laboratory space on a different floor of the building. This precaution was taken to minimize the spread of LAMP amplicon contamination. (Note: The LAMP reaction volume could be at least increased to 100 μL without losing sensitivity.)

### Standard reverse transcription (RT)-LAMP reaction

Mixtures containing different concentrations of MERS-CoV RNA, 1 μM each B1c-B2 and F1c-F2, 0.25 μM each B3 and F3, 0.5 μM LP, 1 M betaine, 2 mM MgCl_2_, and 0.4 mM dNTPs in a total volume of 24 μL 1 × Thermopal buffer NEB; 20 mM Tris-HCl, 10 mM (NH_4_)_2_SO_4_, 10 mM KCl, 2 mM MgSO_4_, 0.1% Triton® X-100, pH 8.8 at 25 °C), 0.5 × AMV RT buffer (NEB; 50 mM Tris-HCl, 75 mM potassium acetate, 8 mM magnesium acetate, 10 mM DTT, pH 8.3 at 25 °C) were heated to 95 °C for 1 min, followed by chilling on ice for 2 min (this pre-denaturing process was not necessary but typically results in improved sensitivity). Then, 1 μL (8 U) of Bst polymerase 2.0 and 0.2 μL (2 U) AMV reverse transcriptase were added to initiate the RT-LAMP reaction. The reactions were incubated for 1.5 hour in a thermal cycler maintained at 55 ^o^C. A 5 μl aliquot of the reaction mixed with 3 μl of a 6 × DNA loading dye was then analyzed by electrophoresis through a 1% agarose gel containing ethidium bromide. Gel analysis of LAMP products was performed in a room completely separate from the normal laboratory space on a different floor of the building. This precaution was taken to minimize the spread of LAMP amplicon contamination. (Note: The RT-LAMP reaction volume could be at least increased to 100 μL without losing sensitivity.)

### LAMP-OSD-glucometer transduction

#### Standard end-point detection

##### Protocol 1 and Protocol 2:

A series of tubes containing 9 μL aliquots of 2 mg/mL Inv-FPc/FP/MBs were placed close to the magnetic rack for 1 min. The clear solution was discarded and replaced by 10 μL standard LAMP or RT-LAMP reaction products (in 1 × Isothermal Buffer) with different starting concentration of templates. The OSD reactions were allowed to proceed for 1 hour on a vertical rotator at 25 °C (Protocol 1) or 55 °C (Protocol 2). The Inv-FPc/FP/MBs were separated using a magnetic rack and 3 μL of the resulting supernatant was transferred into separate tubes containing 3 μL of 500 mM sucrose. This mixture was incubated for 23 min (Protocol 1) and 11 min (Protocol 2) at 55 °C to allow invertase-mediated catalytic conversion of sucrose to glucose. Subsequently, 1 μL of the reaction solution was transferred to a glucometer strip and the glucose concentration was measured by using a commercially available hand-held blood glucometer. Note: All the 25 °C steps were performed on the laboratory bench while the 55 °C steps in Protocol 2 were performed in an incubator maintained at 55 °C.

#### Standard one-tube LAMP-OSD transduction

##### Protocol 3:

A series of tubes containing 9 μL aliquots of 2 mg/mL Inv-FPc/FP/MBs were placed close to the magnetic rack for 1 min. The clear solution was discarded and replaced by 50 μL standard LAMP or RT-LAMP reaction reagents. The one-tube LAMP plus OSD reactions were performed for 1.5 hour on a vertical rotator kept inside an incubator maintained at 55 °C. The Inv-FPc/FP/MBs were then separated using a magnetic rack at 55 °C and 3 μL of the resulting supernatant was transferred into tubes containing 3 μL of 500 mM sucrose. This mixture was incubated for 15 min at 55 °C to allow invertase-mediated catalytic conversion of sucrose to glucose. Subsequently, 1 μL of the reaction solution was transferred to a glucometer strip and the glucose concentration was measured by using a commercially available glucometer.

### Reproducibility and stability tests for Inv-FPc/FP/MBs

The stability and reproducibility tests were mostly performed with Protocol 1. The reproducibility test was carried out by comparing three parallel assays for detecting both “sORF1A positive samples (2E4 copies)” and “buffer negative controls” using Inv-FPc/FP/MBs prepared on three consecutive days, respectively. The stability test was carried out by measuring “sORF1A positive samples (2E4 copies)” every 2 to 20 days for a total of 60 days.

### OR gate platform

The procedures for the preparation of Inv-1A-FPc and Inv-upE-FPc conjugates were the same as that of Inv-FPc. The Inv-1A-FPc/Inv-upE-FPc/OR-P/MBs conjugates were prepared as follows: First, 3 μL of 150 μM biotin-ORP, 5 μL of 5 mg/mL Inv-1A-FPc and 5 μL of 5 mg/mL Inv-upE-FPc were mixed in 18 μL of 2 × Isothermal Buffer. This mixture was heated at 95 °C for 5 min and slowly cooled to room temperature at a rate of 0.1 °C/s to obtain the Inv-1A-FPc/Inv-upE-FPc/OR-P complex. Second, 200 μL of 1 mg/mL streptavidin-coated MBs were transferred into a 1.5 mL centrifuge tube and washed in 1x isothermal amplification buffer with the aid of an external magnetic rack. The resulting MBs were resuspended in 50 μL 1 × Isothermal Buffer and then incubated for 25 min with 36 μL of Inv-1A-FPc/Inv-upE-FPc/OR-P solution on a vertical rotator. Following five washes with 100 μL isothermal amplification buffer to remove excess Inv-1A-FPc/Inv-upE-FPc/OR-P, the Inv-1A-FPc/Inv-upE-FPc/OR-P/MBs conjugates were resuspended and stored at 4 °C for further use.

OR gated LAMP-to-Glucometer detection was performed using Protocol 1 as detailed above. RT-LAMP amplicons were generated from the same amount of MERS-CoV RNA (60 PFU) by using either no primer, upE primer (upE.9) only, 1A primer (ORF1A.55) only, or both upE.9 and ORF1A.55 primers, respectively.

### Detection of Zaire Ebolavirus (ZEBOV) using LAMP-to-glucose transduction

ZEBOV detection in buffer was realized with Protocol 1, by using ZEBOV VP30 synthetic target and by replacing MERS-CoV-specific reagents with ZEBOV.3.3 LAMP primer set (containing FIP, BIP, F3 and B3) and the ZEBOV-FP/Inv-ZEBOV-FPc probe complex. For ZEBOV detection in complex samples, different amounts of the ZEBOV VP30 synthetic template (in H_2_O) was freshly added into treated synthetic sputum or human saliva before use. For each 25 μL LAMP reaction, the mixture contained 6.5 μL of sputum or 5 μL of saliva, and the final volume ratio of sputum and saliva in each reaction was therefore 26% and 20%, respectively.

### Synthetic sputum and human saliva samples

Synthetic sputum was provided by PATH (Program for Appropriate Technology in Health, PATH, Seattle, WA) and consisted of 47 mg/mL porcine mucin, 6 mg/mL salmon sperm DNA, 3.6 mg/mL phosphatidylcholine and 33 mg/mL bovine serum albumin in 114 mM NaCl, 2 mM sodium azide. Before use, synthetic sputum was thawed and the mucin was liquefied with 2% freshly made N-acetyl-L-cysteine and 1% sodium hydroxide for 15 min at room temperature. Following digestion the contents were diluted 50-fold with phosphate buffered saline, centrifuged at 3000 × rcf for 20 min, and the supernatant discarded. The resulting pellet was resuspended in 300 μL TE buffer (10 mM Tris, 1 mM EDTA, pH 8.0). After freezing at −80 ^o^C and thawing at 95 ^o^C over 5 cycles, the solution was centrifuged at 1600 × rcf for 10 min and the supernatant was collected and stored at −20 ^o^C. Before the LAMP reaction, the supernatant was thawed on ice and 13 μL was mixed with 1 μL of different amounts of ZEBOV VP30 synthetic template; approximately half of this sample was used in the LAMP reaction.

Saliva was directly collected from one of our lab members, following a 10 hour fast. A 1 mL sterile syringe (BD, Franklin Lakes, NJ) with no needle was inserted under the tongue and slowly pulled to collect ~100 μL saliva. Following collection, the saliva sample was heated at 95 ^o^C for 10 min. After cooling to room temperature, a 10 μL sample was mixed with 1 μL of different amounts of ZEBOV VP30 synthetic template; approximately half of this sample was used in the LAMP reaction.”

## Additional Information

**How to cite this article**: Du, Y. *et al.* A Sweet Spot for Molecular Diagnostics: Coupling Isothermal Amplification and Strand Exchange Circuits to Glucometers. *Sci. Rep.* **5**, 11039; doi: 10.1038/srep11039 (2015).

## Supplementary Material

Supplementary Information

## Figures and Tables

**Table 1:** **Sequences of oligonucleotides used in this work (5’ to 3’)**

ORF1A.55 primer set	F3^a^	TTATGCAAACATAGTCTACGAG
	B3	CGCAAAGTTAGAAAGTGATGG
	FIP	AAGCATTAGTGGGGGCAAGCCCCACTACTCCCATTTCG
	BIP	ATGCGCACTACACATACTGATATTTGTACAATCTCTTCACTACAATGA
	LP	GGTGTCTACATTAGTATGTCACTTGTATTAG
sORF1AOSD probe set	FP	CGAAGCCAATTTGCAACTGCAATCAGCGCTGAG AAAAAAAAA /3’Biotin/
	FPc	ATTGCAGTTGCAAATTGGCTTCG AAAAAAAAAAAA /3’Thiol/
upE.9primer set	F3	AGTAAGATTAGCCTAGTTTCTGT
	B3	TCCATATGTCCAAAGAGAGAC
	FIP	GAGGAACTGAATCGCGCGTTGACTTCTCCTTAAACGGCA
	BIP	TTCACATAATCGCCCCGAGCTAATGGATTAGCCTCTACACG
	LP	GCAGGCACGAAAACAGTGGAAACAT
ORGateOSD probe set	ORP	/5Biosg/AAAAAAAAAATCGCTTATCGTTTAAGCAGCTCTGCGCTAC TATGG GTCC CGAAGCCAATTTGCAACTGCAATCAGCGCTGAGC
	1A-FPc	/5ThioMC6-D/ AAAAAAAAAAAA ATTGCAGTTGCAAATTGGCTTCG
	upE-FPc	/5ThioMC6-D/AAAAAAAAAAAA TAGCGCAGAGCTGCTTAAACGATAAGCGA
Mimical target	1A-T	CTCAGCGCTGATTGCAGTTGCAAATTGGCTTCG
	upE-T	GGACCCATAGTAGCGCAGAGCTGCTTAAACGATAAGCGA
	Random sequence (or Random C)	CGACATCTAACCTAGCTCACTGAC’
ZEBOV. 3.3 primer set	F3	AGACAGCATTCAAGGGATG
	B3	CCTTTTTTCAAGGTCGGACA
	FIP	CTCCTTGATTGACGGTACTCACCGACACGACCACCATGTTC
	BIP	CTCACAAGTGCGCGTTCCTAATGTCTTTAGGTGCTGGAG
ZEBOV OSD probe set	ZEBOV-FP	GAGCACGATCATCATCCAGAGAGAATTATCGAGGTGCA AAAAAAAAAA/3’Biotin/
	ZEBOV-FPc	TGCACCTCGATAATTCTCTCTGGATGAT AAAAAAAAAAAA /3’Thiol/

^a^F3: forward outer primer, B3: reverse outer primer, FIP: forward inner primer, BIP: reverse inner primer, LP: loop primer

## References

[b1] Zaki A. M., van Boheemen S., Bestebroer T. M., Osterhaus A. D. M. E. & Fouchier R. A. M. Isolation of a Novel Coronavirus from a Man with Pneumonia in Saudi Arabia. New Engl. J. Med. 367, 1814–1820 (2012). 23075143 10.1056/NEJMoa1211721

[b2] Holmes D. MERS-CoV enigma deepens as reported cases surge. Lancet 383, 1793–1793 (2014). 24868566 10.1016/S0140-6736(14)60866-7 PMC7137153

[b3] Cotten M. *et al.* Transmission and evolution of the Middle East respiratory syndrome coronavirus in Saudi Arabia: a descriptive genomic study. Lancet 382, 1993–2002(2013). 24055451 10.1016/S0140-6736(13)61887-5 PMC3898949

[b4] Update: Recommendations for Middle East Respiratory Syndrome Coronavirus (MERS-CoV). Mmwr-Morbid Mortal. W. 62, 557–557 (2013). PMC4604945 23842446

[b5] Bialek S. R. *et al.* First Confirmed Cases of Middle East Respiratory Syndrome Coronavirus (MERS-CoV) Infection in the United States, Updated Information on the Epidemiology of MERS-CoV Infection, and Guidance for the Public, Clinicians, and Public Health Authorities - May 2014. Mmwr-Morbid Mortal. W. 63, 431–436 (2014). PMC5779407 24827411

[b6] Coleman C. M. & Frieman M. B. Coronaviruses: Important Emerging Human Pathogens. J. Virol. 88, 5209–5212 (2014). 24600003 10.1128/JVI.03488-13 PMC4019136

[b7] Corman V. M. *et al.* Assays for laboratory confirmation of novel human coronavirus (hCoV-EMC) infections. Eurosurveill. 17, pii:20334 (2012). 10.2807/ese.17.49.20334-en 23231891

[b8] Corman V. M. *et al.* Detection of a novel human coronavirus by real-time reverse-transcription polymerase chain reaction. Eurosurveill.17, pii: 20285 (2012). 10.2807/ese.17.39.20285-en 23041020

[b9] Fauci A. S. An audience with - Anthony Fauci. Nat. Rev. Drug. Discov. 7, 12–12 (2008).

[b10] Corman V. M. *et al.* Performance and clinical validation of the RealStar (R) MERS-CoV Kit for detection of Middle East respiratory syndrome coronavirus RNA. J. Clin. Virol. 60, 168–171(2014). 24726679 10.1016/j.jcv.2014.03.012 PMC7106532

[b11] Compton J. Nucleic-Acid Sequence-Based Amplification. Nature 350, 91–92 (1991). 1706072 10.1038/350091a0

[b12] Dean F. B. *et al.* Comprehensive human genome amplification using multiple displacement amplification. Proc. Natl. Acad. Sci. USA 99, 5261–5266 (2002). 11959976 10.1073/pnas.082089499 PMC122757

[b13] Hall M. J., Wharam S. D., Weston A., Cardy D. L. N. & Wilson W. H. Use of signal-mediated amplification of RNA technology (SMART) to detect marine cyanophage DNA. Biotechniques 32, 604–6 (2002). 11926174 10.2144/02323rr02

[b14] Walker G. T. *et al.* Strand Displacement Amplification - an Isothermal, Invitro DNA Amplification Technique. Nucleic Acids Res. 20, 1691–1696 (1992). 1579461 10.1093/nar/20.7.1691 PMC312258

[b15] Kurn N. *et al.* Novel isothermal, linear nucleic acid amplification systems for highly multiplexed applications. Clin. Chem. 51, 1973–1981 (2005). 16123149 10.1373/clinchem.2005.053694

[b16] Crannell Z. A., Castellanos G. A., Irani A., Rohrman B., White A. C. & Richard. K. R. Nucleic Acid Test to Diagnose Cryptosporidiosis: Lab Assessment in Animal and Patient Specimens. Anal. Chem. 86, 2565–2571 (2014). 24479858 10.1021/ac403750z PMC3958140

[b17] Notomi T. *et al.* Loop-mediated isothermal amplification of DNA. Nucleic Acids Res. 28, e63(2000). 10871386 10.1093/nar/28.12.e63 PMC102748

[b18] Nagamine K., Kuzuhara Y. & Notomi T. Isolation of single-stranded DNA from loop-mediated isothermal amplification products. Biochem. Bioph. Res. Co. 290, 1195–1198 (2002). 10.1006/bbrc.2001.6334 11811989

[b19] Li B. L., Chen X. & Ellington A. D. Adapting Enzyme-Free DNA Circuits to the Detection of Loop-Mediated Isothermal Amplification Reactions. Anal. Chem. 84, 8371–8377 (2012). 22947054 10.1021/ac301944v PMC3478682

[b20] Bhadra S. *et al.* Real-time sequence-validated loop-mediated isothermal amplification assays for detection of Middle East respiratory syndrome coronavirus (MERS-CoV). PlosOne, just accepted (2015). 10.1371/journal.pone.0123126 PMC4391951 25856093

[b21] Jiang Y. S. *et al.* Robust strand exchange reactions for the sequence-specific, real-time detection of nucleic acid amplicons, Anal. Chem. 87, 3314–3320 (2015). 25708458 10.1021/ac504387c

[b22] Xiang Y. & Lu Y. Using personal glucose meters and functional DNA sensors to quantify a variety of analytical targets. Nat. Chem. 3, 697–703 (2011). 21860458 10.1038/nchem.1092 PMC3299819

[b23] Xiang Y. & Lu Y. An invasive DNA approach toward a general method for portable quantification of metal ions using a personal glucose meter. Chem. Commun. 49, 585–587(2013). 10.1039/c2cc37156a PMC3765066 23208450

[b24] Xiang Y. & Lu Y. Using Commercially Available Personal Glucose Meters for Portable Quantification of DNA. Anal. Chem. 84, 1975–1980 (2012). 22235863 10.1021/ac203014s PMC3302979

[b25] Zhou Z. J., Xiang Y., Tong A. J. & Lu Y. Simple and Efficient Method to Purify DNA-Protein Conjugates and Its Sensing Applications. Anal. Chem. 86, 3869–3875 (2014). 24605905 10.1021/ac4040554 PMC4004194

[b26] Liao J. Y. & Li H. Target-induced DNAzyme Cleavage Accompanying Bioactive Enzymatic Assembly with Glucometer Readout for Quantitative Monitoring of Lead Ion. Chem. Lett. 43, 1599–1600 (2014).

[b27] Joo J. *et al.* A facile and sensitive method for detecting pathogenic bacteria using personal glucose meters. Sensor Actuat. B-Chem., 188, 1250–1254 (2013).

[b28] Wang Q. *et al.* Multiplex detection of nucleic acids using a low cost microfluidic chip and a personal glucose meter at the point-of-care. Chem. Commun., 50, 3824–3826 (2014). 10.1039/c4cc00133h 24590123

[b29] Hun X., Xu Y. Q., Xie G. L. & Luo X. L. Aptamer biosensor for highly sensitive and selective detection of dopamine using ubiquitous personal glucose meters. Sensor Actuat. B-Chem., 209, 596–601 (2015).

[b30] Xu X. T., Liang K. Y. & Zeng J. Y. Portable and sensitive quantitative detection of DNA based on personal glucose meters and isothermal circular strand-displacement polymerization reaction. Biosens. Bioelectron., 64, 671–675 (2015). 25441417 10.1016/j.bios.2014.09.094

[b31] WHO. Laboratory Testing for Middle East Respiratory Syndrome Coronavirus. Available: http://www.who.int/csr/disease/coronavirus_infections/WHO_interim_recommendations_lab_detection_MERSCoV_092014.pdf?ua=1. Accessed 5 March 2015.

[b32] M Citri N. & Zyk N. The interaction of penicillinase with penicillins IV. Structural aspects of catalytic and non-catalytic interactions. Biochimica Et Biophysica Acta 99, 427–441 (1965). 5840962 10.1016/s0926-6593(65)80197-7

[b33] Kuramits H. k. Characterization of Invertase Activity from Cariogenic Streptococcus mutans. J. Bacteriol. 115, 1003–1010 (1973). 4353868 10.1128/jb.115.3.1003-1010.1973 PMC246348

[b34] Griessler R., D’Auria S., Tanfani F. & Nidetzky B. Thermal denaturation pathway of starch phosphorylase from Corynebacterium callunae: Oxyanion binding provides the glue that efficiently stabilizes the dimer structure of the protein. Protein Sci. 9, 1149–1161 (2000). 10892808 10.1110/ps.9.6.1149 PMC2144666

[b35] Zhu J. B., Zhang L. B., Li T., Dong S. J. & Wang E. K. Enzyme-Free Unlabeled DNA Logic Circuits Based on Toehold-Mediated Strand Displacement and Split G-Quadruplex Enhanced Fluorescence. Adv. Mater. 25, 2440–2444 (2013). 23447454 10.1002/adma.201205360

[b36] Teichmann M., Kopperger E. & Simmel F. C. Robustness of Localized DNA Strand Displacement Cascades. Acs Nano 8, 8487–8496 (2014). 25089925 10.1021/nn503073p

[b37] Liu M. *et al.* A DNA tweezer-actuated enzyme nanoreactor. Nat. Commun. 4, 2127 (2013). 23820332 10.1038/ncomms3127

[b38] Drosten C. *et al.* Clinical features and virological analysis of a case of Middle East respiratory syndrome coronavirus infection. Lancet Infect. Dis. 13, 745–751 (2013). 23782859 10.1016/S1473-3099(13)70154-3 PMC7164791

[b39] Center for Dessease control and prevention: First Confirmed Cases of Middle East Respiratory Syndrome Coronavirus (MERS-CoV) Infection in the United States, Updated Information on the Epidemiology of MERS-CoV Infection, and Guidance for the Public, Clinicians, and Public Health Authorities, MMWR, 63, 431–436 (2014). PMC5779407 24827411

[b40] Sashikumar R. & Kannan R. Salivary glucose levels and oral candidal carriage in type II diabetics. Oral Surg. Oral Med. Oral Pathol. Oral. Radiol. Endod. 109, 706–711(2010). 20416536 10.1016/j.tripleo.2009.12.042

[b41] Bausch D. G. *et al.* Assessment of the risk of Ebola virus transmission from bodily fluids and fomites. J. Infect. Dis. 196, S142–S147, 10.1086/520545 (2007). 17940942

[b42] Formenty P. *et al.* Detection of Ebola virus in oral fluid specimens during outbreaks of Ebola virus hemorrhagic fever in the republic of Congo. Clin. Infect. Dis. 42, 1521–1526 (2006). 16652308 10.1086/503836

[b43] Richardson S. M., Wheelan S. J., Yarrington R. M. & Boeke J. D. GeneDesign: Rapid, automated design of multikilobase synthetic genes. Genome Res. 16, 550–556 (2006). 16481661 10.1101/gr.4431306 PMC1457031

[b44] Gibson D. G. *et al.* Enzymatic assembly of DNA molecules up to several hundred kilobases. Nat. Methods 6, 343–341 (2009). 19363495 10.1038/nmeth.1318

[b45] Cox J. C., Lape J., Sayed M. A. & Hellinga H. W. Protein fabrication automation. Protein Science 16, 379–390 (2007). 17242375 10.1110/ps.062591607 PMC2203321

