# Supplementary material for: A Sweet Spot for Molecular Diagnostics: Coupling Isothermal Amplification and Strand Exchange Circuits to Glucometers
Source: Sci Rep. 2015 Jun 8;5:11039. doi: 10.1038/srep11039 (PMC4458886; doi:10.1038/srep11039)
Supplement: Supplementary Information [file srep11039-s1.pdf]

Supporting Information

A Sweet Spot for Molecular Diagnostics: Coupling Isothermal Amplification and Strand Exchange Circuits to Glucometers

Yan Du[a], Randall A. Hughes[b], Sanchita Bhadra[a], Yu Sherry Jiang[c], Andrew D. Ellington*[a], Bingling Li*[a]

[a] Center for Systems and Synthetic Biology, The University of Texas at Austin, Austin, TX 78712, USA
[b]Applied Research Laboratories, The University of Texas at Austin, Austin, TX 78758, USA
[c]Department of Chemsitry, The University of Texas at Austin, Austin, TX 78712, USA

Email: binglingli1982@gmail.com; andy.ellington@mail.utexas.edu

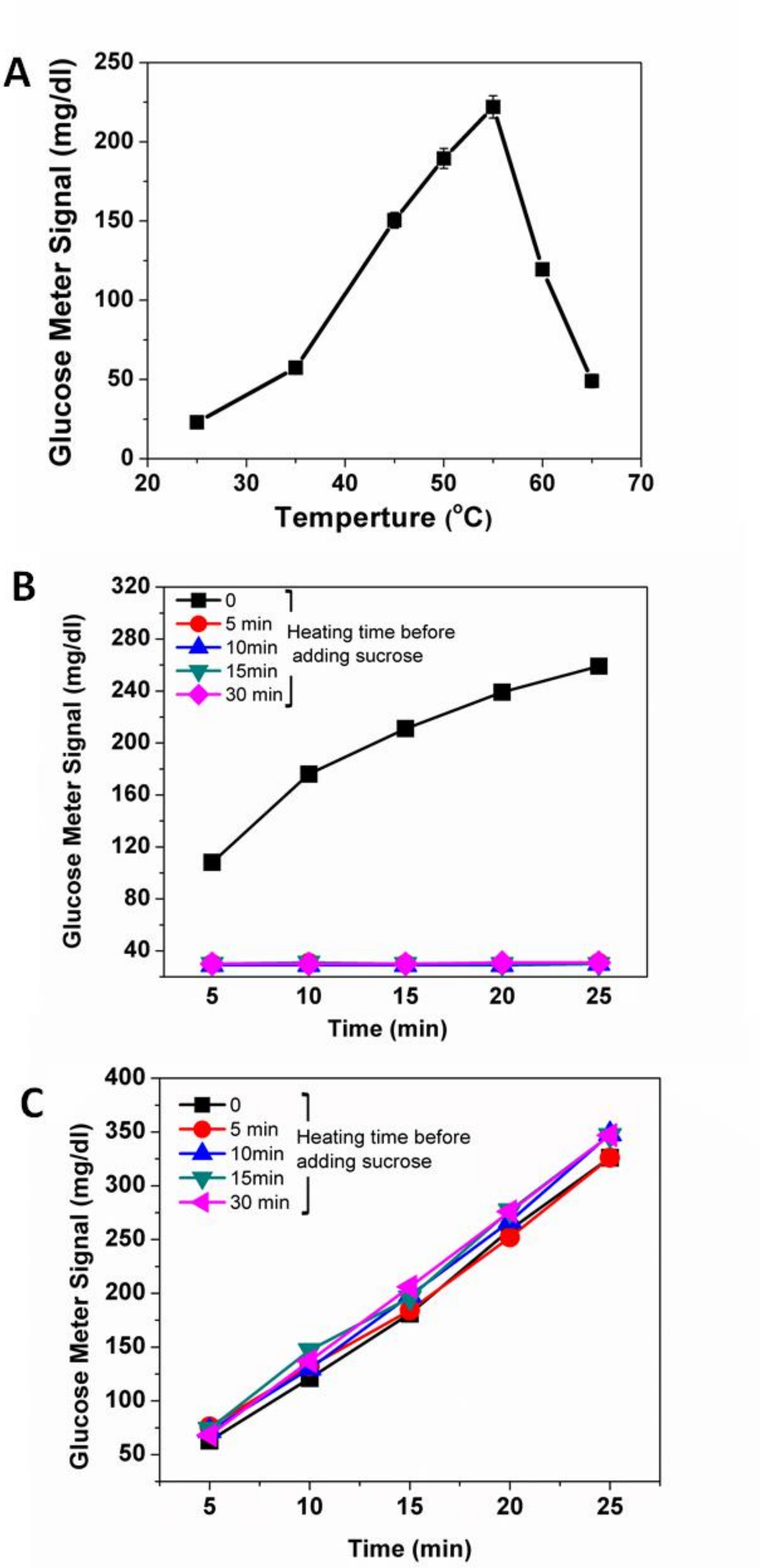

A
Glucose Meter Signal (mg/dl)
250
200
150
100
50
0
20
30
40
50
60
70
Temperture (°C)
B
Glucose Meter Signal (mg/dl)
320
280
240
200
160
120
80
40
0
5 min
10min
15min
30 min
Heating time before
adding sucrose
5
10
15
20
25
Time (min)
C
Glucose Meter Signal (mg/dl)
400
350
300
250
200
150
100
50
0
5 min
10min
15min
30 min
Heating time before
adding sucrose
5
10
15
20
25
Time (min)

Figure S1**:** (A) **Temperature dependence of yeast invertase-FPc conjugate (Inv-FPc) activity**. At each temperature, a 1.8 uL mixture containing 1.8 μg Inv-FPc/FP/MBs and 250 mM sucrose was incubated for 3 min, followed by measurement of glucose yielded using a glucometer. (B) **Time dependence of yeast invertase inactivation.** After pre-heating at 55 ^{o}C for variable periods of time (0 min, 5 min, 10 min, 15 min, and 30 min) in the absence of sucrose, 50 μg/mL yeast invertase (270 kDa) was mixed with 500 mM sucrose at a 1:1 volume ratio and further incubated at 55 ^{o}C, followed by a glucometer measurement every 5 min. (C) **Time dependence of thermostable yeast invertase (TmINV) inactivation.** Experiments were similar to those shown for Figure S1B, except that 10 μg/mL thermostable invertase (55 kDa) was used. This represents an equimolar concentration (mole/L) of invertase as in **Figure S1B**. For all experiments in **Figures S1B** and **S1C**, 25 mg/dl glucose was also manually spiked into the sucrose solution to guarantee there was a readable signal on the glucometer.

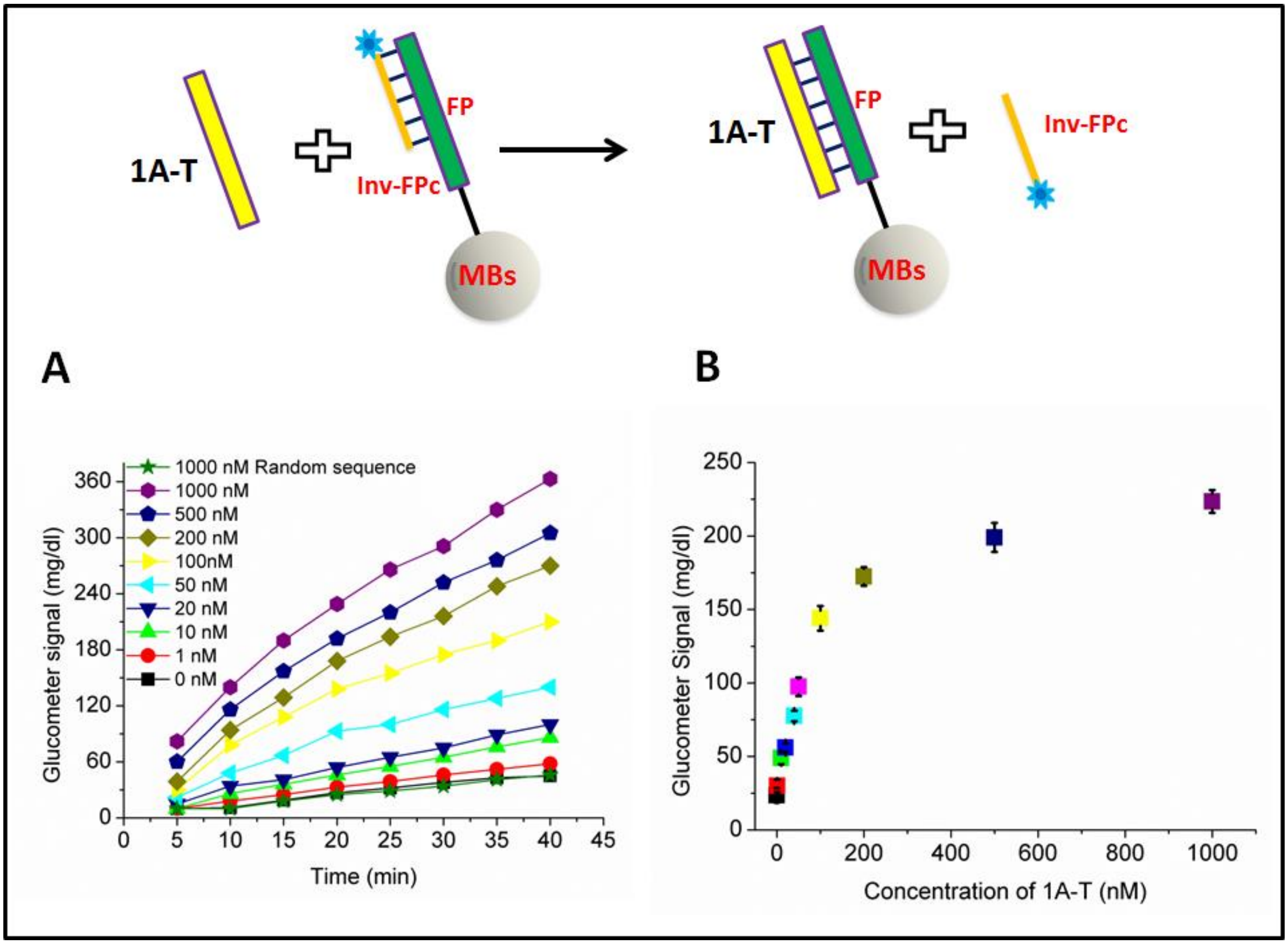

Figure S2. **Detection of DNA mimic of the LAMP loop (1A-T) using the OSD-to-glucose transduction.** 1A-T directly initiates a one-step strand displacement (OSD) reaction to release the Inv-FPc reporter sequence from the capture FP sequence conjugated to magnetic beads. After magnetic separation, the released Inv-FPc reporter sequence is then detected by the production of glucose by the conjugated invertase and readout via a glucometer. (A) Kinetic curves of glucose generation in presence of different concentrations of 1A-T and 1 μM non-specific sequence (Random sequence). (B) Concentration dependence of 1A-T using the 40 min glucometer signal collected from **Figure S2A**. For these assays a commercially available yeast invertase was used with 1 hour 25 ^{o}C OSD.

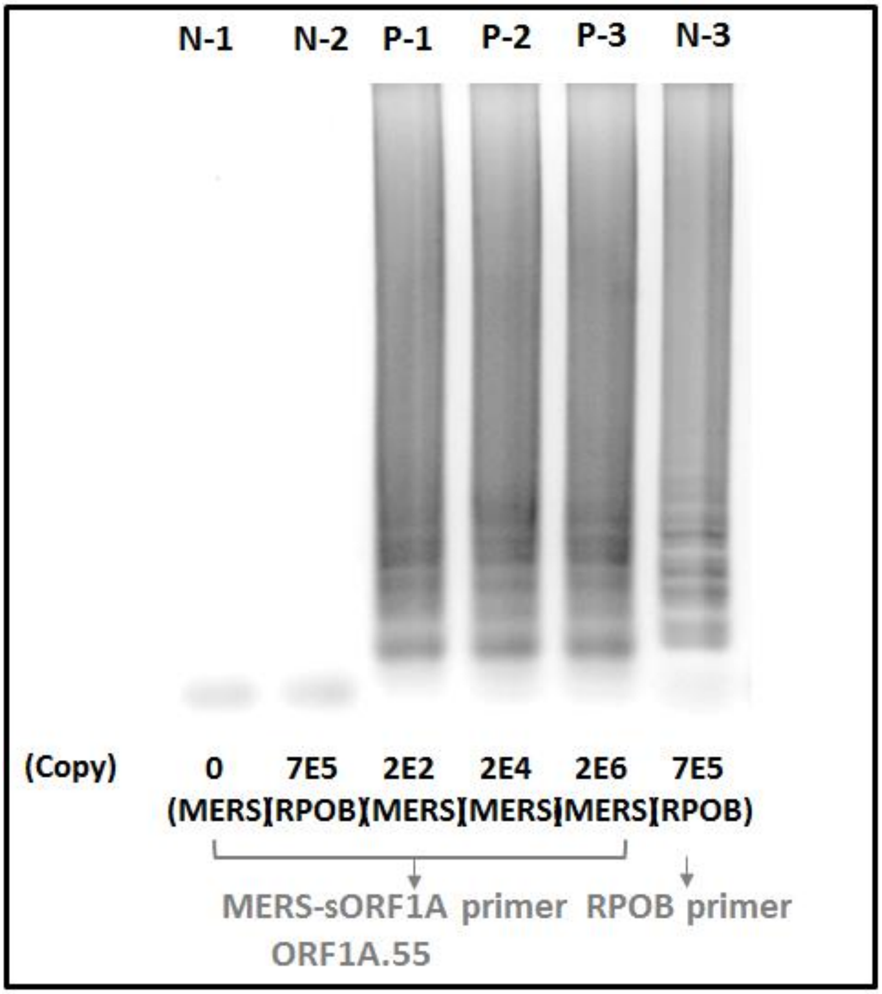

***Figure S3*. Agarose gel electrophoretic characterization of LAMP amplicons produced from target and non-target templates.** N-1, N-2, P-1, P-2, P-3 represent LAMP reactions amplified from 2E2 copies, 2E4 copies, and 2E6 copies of MERS-CoV sORF1A DNA (=MERS), buffer control, and 7E5 copies of non-specific target control (RPOB), in presence of the MERS-CoV specific primer set, OSF1A.55. N-3 represents LAMP reaction amplified from 7E5 copies of non-specific target control (RPOB) in presence of a RPOB specific primer set. For each sample, LAMP reaction is carried out at 55 ^{o}C for 1.5 hour. Then a 5 µl aliquot of the each reaction mixed with 3 µl of 6 × DNA loading dye is analyzed by electrophoresis through a 1% agarose gel containing ethidium bromide.

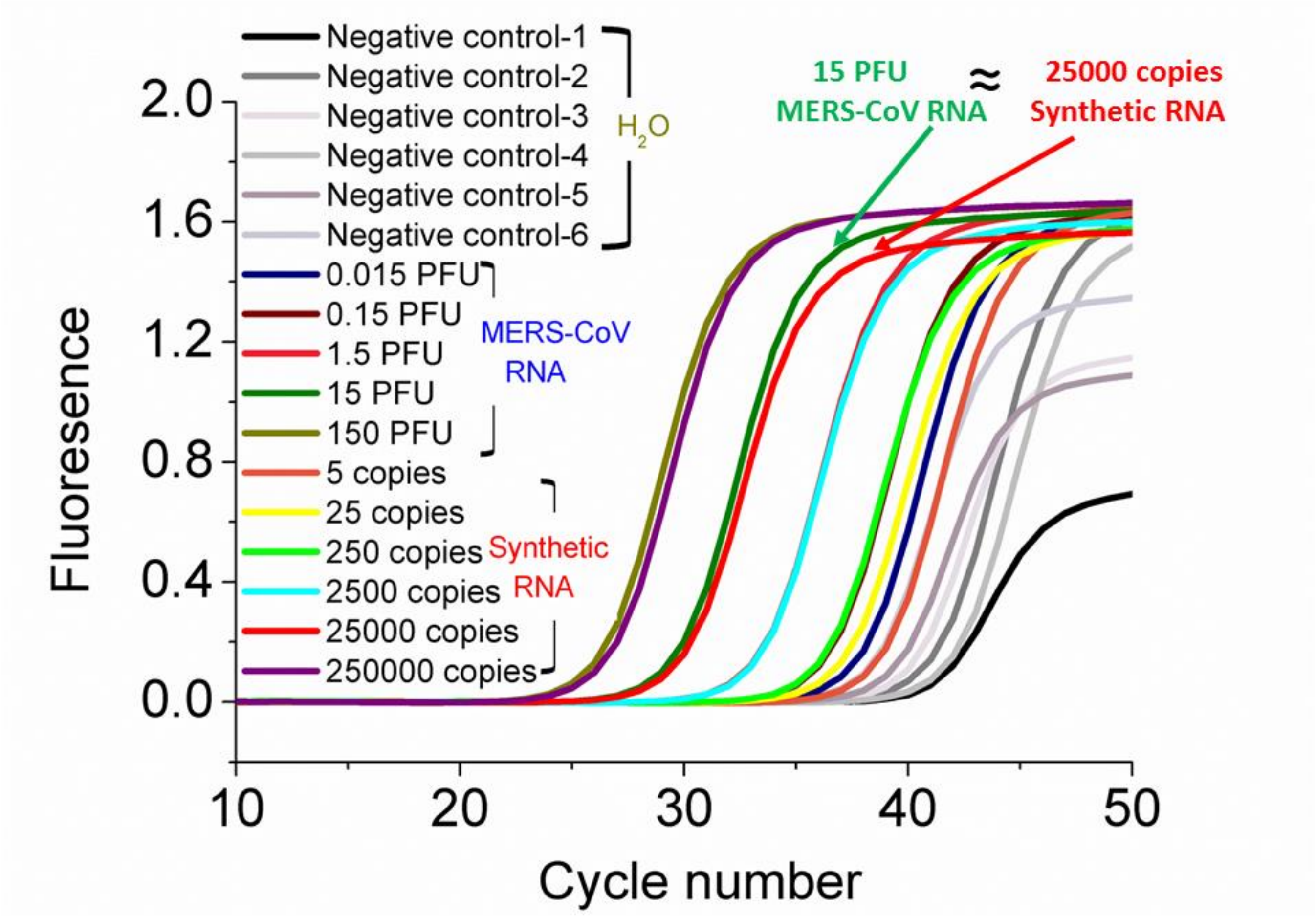

***Figure S4*. Real-time reverse transcription PCR (rtRT-PCR) detection of MERS-CoV RNA.** Templates were either RNA extracted from tissue culture-derived virions or 384-mer *in vitro* transcribed RNA templates (synthetic RNA)[1]. The PCR reactions were set up following the WHO recommended protocol. Each 25 µL reaction contained 0.4 µM reverse and forward primers, 0.2 µM Taqman probe, 1 µL of a SuperScript® III reverse transcriptase and Platinum® Taq DNA polymerase mixture (provided in the Invitrogen superScriptIII OneStep RT-PCR system), 0.2 mM dNTP mixtures, and different amounts of MERS-CoV RNA or synthetic RNA. The reaction conditions were as follows: 55 ^{o}C for 20s, 94 ^{o}C for 3s, followed by 50 cycles of 94 ^{o}C for 15s and annealing/extension at 58 ^{o}C for 30s.

The synthetic RNAs with known copy numbers were to quantify the extracted MERS-CoV RNAs. The equivalence was approximately 25,000 copies of synthetic RNA equals 15 PFU MERS-CoV RNA.

The sequences of the primers used were:

Reverse primer: 5’-CCACTACTCCCATTTCGTCAG-3’

Forward primer: 5’-CAGTATGTGTAGTGCGCATATATGCA-3’

Taqman probe: 5’-FAM-TTGCAAATTGGCTTGCCCCCACT-TAMRA-3’

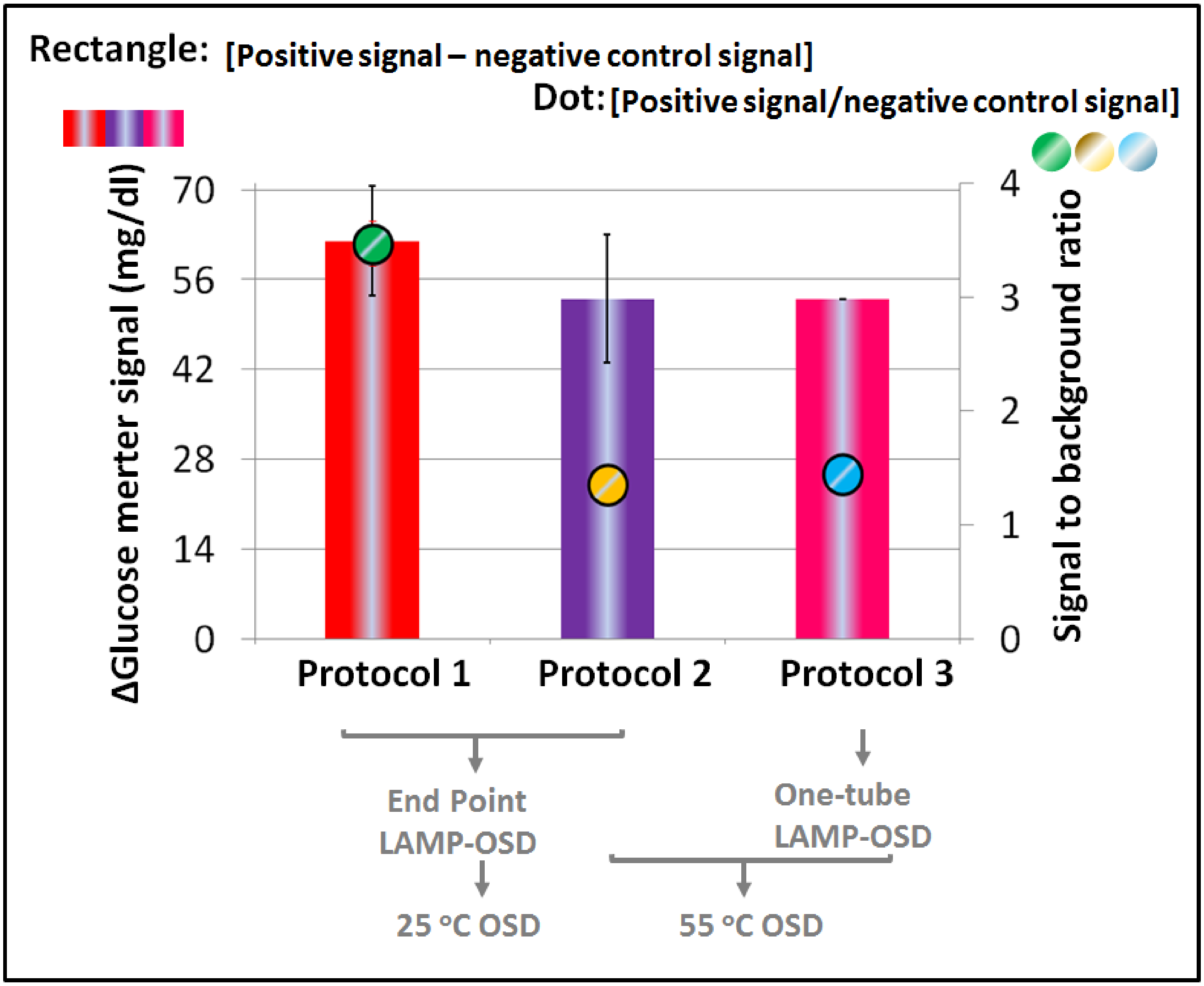

***Figure S5*. Realization of high temperature OSD and one-tube LAMP-OSD transduction.** The graph shows the signal amplitude (ΔGlucose meter signal, positive signal – negative signal, left y-axis, rectangles) and signal-to-background ratio (positive signal / negative signal, right y-axis, dots) of the three sensing protocols. Positive signal is glucometer response to RNA extracted from 60 PFU MERS-CoV. Negative signal is glucometer response to buffer negative control. Protocol 1: 1.5 hour 55 ^{o}C RT-LAMP, 1 hour 25 ^{o}C OSD, and 23 min 55 ^{o}C glucose generation is carried out in a sequential pathway. Protocol 2: 1.5 hour 55 ^{o}C RT-LAMP, 1 hour 55 ^{o}C OSD, and 11 min 55 ^{o}C glucose generation is carried out in a sequential pathway. Protocol 3: RT-LAMP and OSD is carried out simultaneously (in one tube) at 55 ^{o}C for 1.5 hour, followed by 15 min 55 ^{o}C glucose generation. The thermostable TmINV was used in all these experiments.

500 nM UpE-T	500 nM 1A-T	Output
0	0	0
1	0	1
0	1	1
1	1	1

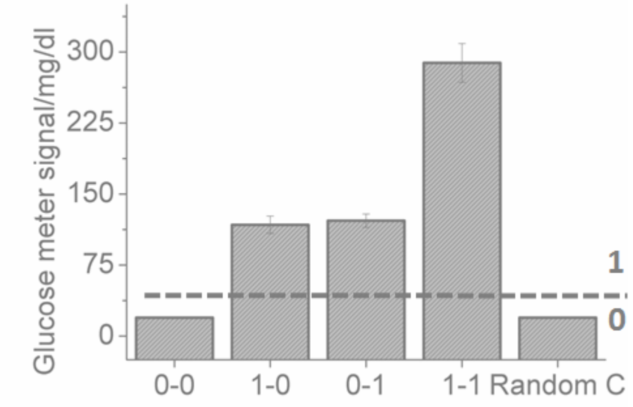

Figure ***S6*****. OR gate glucometer responses to DNA mimics of the LAMP loops (1A-T and upE-T) amplified from two regions on the MERS-CoV genome.** (A) True value table of OR gate with 1A-T and UpE-T as two inputs. (B) OR gate glucometer responses to buffer control (0-0), 500 nM 1A-T (0-1), 500 nM upE-T (1-0), 500nM 1A-T and upE (1-1) mixture, and a 1 μM non-specific input (Random C) control. The thermostable TmINV was used in these experiments, with a 1.5 hour 55 ^{o}C RT-LAMP, 1 hour 25 ^{o}C OSD, and 23 min 55 ^{o}C glucose generation cycle.

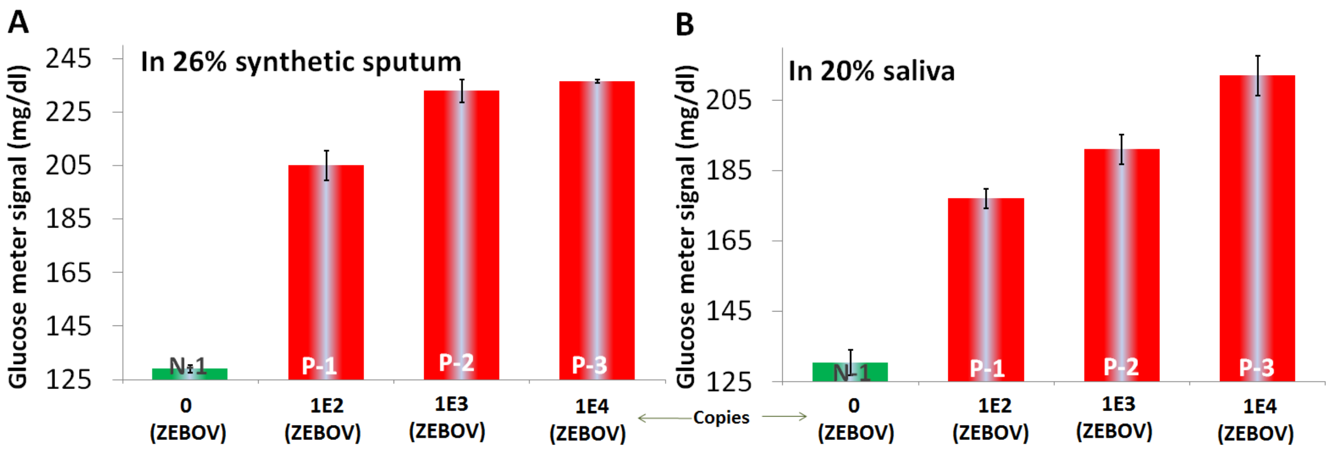

***Figure S7.* Detection of synthetic ZEBOV DNA (ZEBOV-VP30) using LAMP-to-glucose transduction in synthetic sputum or human saliva.** Glucometer responses to 1E2 copies (P-1), 1E3 copies (P-2) and 1E4 copies (P-3) of synthetic ZEBOV VP30 (= ZEBOV) or to a DNA buffer control (0 copies, N-1) in the presence of (A) 26% treated mucin synthetic sputum or (B) 20% treated human saliva. Reaction conditions were as in **Figure 3**. The error bars represent standard deviations calculated from two parallel assays. The thermostable TmINV was used in these experiments.

References:

1. Bhadra, S. *et al.* Real-time sequence-validated loop-mediated isothermal amplification assays for detection of Middle East respiratory syndrome coronavirus (MERS-CoV). *PlosOne*, **just accepted** (2015).
